# Endoscopic Hemostasis as a Bridge to Surgical Portal Decompression in Children with Portal Hypertensive Bleeding: A Staged Endoscopic–Surgical Strategy

**DOI:** 10.3390/children13050624

**Published:** 2026-04-30

**Authors:** Jianji Xu, Jinshan Zhang, Chihuan Kong

**Affiliations:** Department of General Surgery, Capital Institute of Pediatrics, Capital Medical University, Beijing 100020, China; xujianjicip@outlook.com

**Keywords:** portal hypertension, pediatric, variceal bleeding, endoscopic therapy, Rex shunt, bridging therapy

## Abstract

**Highlights:**

**What are the main findings?**

- A staged endoscopic–surgical strategy appears feasible for achieving early stabilization and timely portal decompression in children with acute portal hypertensive bleeding.
- Early Rex shunt dysfunction (<3 months) was observed in a small subgroup of children younger than 3 years.

**What are the implications of the main findings?**

- These findings suggest a possible age-related signal in early shunt dysfunction, which should be interpreted cautiously.
- The results support further multicenter studies to refine patient selection and optimize the timing of surgical intervention.

**Abstract:**

**Background:** Portal hypertension is a major cause of esophagogastric variceal bleeding in children. Endoscopic therapy is widely used for acute hemostasis; however, it primarily controls the bleeding episode rather than the underlying portal hypertensive physiology, and definitive management often requires surgical portal decompression. Evidence regarding the outcomes of a staged endoscopic–surgical management strategy in pediatric patients remains limited. This study aimed to evaluate the clinical outcomes of children with portal hypertensive bleeding managed with endoscopic hemostasis as a bridging therapy followed by definitive portal decompression surgery. **Methods:** We conducted a retrospective consecutive cohort study including 12 children presenting with portal hypertension-related variceal bleeding at our tertiary pediatric center between January 2021 and December 2024. All patients underwent endoscopic hemostasis, followed by evaluation for portal decompression surgery when anatomically feasible. Clinical outcomes including hemostasis success, rebleeding, shunt patency, and survival were analyzed. An age-stratified exploratory analysis was performed to examine the association with early dysfunction after Rex shunt reconstruction. **Results:** Endoscopic hemostasis was successfully achieved in all patients, with no early rebleeding prior to surgery. Ten patients underwent portal decompression surgery within 7 days (Rex shunt, *n* = 8; splenorenal shunt, *n* = 2). During a median follow-up of 18 months, early Rex shunt dysfunction (<3 months) was observed in 2 of 8 patients (25%), both of whom were younger than 3 years, whereas no dysfunction was observed in older children. Given the small sample size, this observation should be interpreted descriptively. Rebleeding and mortality occurred exclusively in association with shunt dysfunction. **Conclusions**: A staged endoscopic–surgical strategy appears feasible for stabilizing children with acute portal hypertensive bleeding and enabling timely definitive portal decompression. In this small cohort (*n* = 12), an age-related signal in early Rex shunt dysfunction was observed in very young children; however, this finding should be interpreted cautiously and requires further validation in larger studies.

## 1. Introduction

Pediatric portal hypertension is an important cause of upper gastrointestinal bleeding and may result in life-threatening esophagogastric variceal hemorrhage [1,2,3]. In children, portal hypertension may result from both extrahepatic portal vein obstruction and intrahepatic disorders such as congenital hepatic fibrosis or biliary atresia. Among these, extrahepatic portal vein obstruction is one of the most common etiologies and frequently leads to the development of esophageal and gastric varices [4]. The clinical consequences of pediatric portal hypertension extend beyond acute bleeding and may include hypersplenism, growth impairment, and long-term hepatobiliary complications. Because of the heterogeneous etiologies and complex hemodynamic alterations involved, the management of portal hypertensive bleeding in children remains clinically challenging.

Endoscopic therapy plays a central role in the acute management of variceal bleeding [5,6]. Techniques such as endoscopic variceal ligation and cyanoacrylate injection are widely used to achieve rapid hemostasis, particularly for high-flow gastric fundal varices [7,8]. Cyanoacrylate injection is effective for achieving rapid hemostasis; however, it carries a potential risk of embolization, particularly in high-flow varices. Coil-assisted embolization has been described as an alternative strategy; however, its application in pediatric patients may be limited by small vessel size and technical feasibility [9]. However, endoscopic treatment primarily addresses the bleeding event rather than the underlying portal hypertensive physiology. Endoscopic therapy therefore functions primarily as a bridging strategy that stabilizes acute bleeding and allows time for evaluation of definitive therapeutic options.

Among surgical options, the Rex shunt (meso-Rex bypass), defined as a mesenteric vein-to-left portal vein bypass performed at the Rex recess, is regarded as the preferred physiologic reconstruction for children with extrahepatic portal vein obstruction because it restores hepatopetal portal flow to the liver [3]. Previous studies have reported favorable long-term outcomes and restoration of physiologic portal venous flow following this procedure [10,11]. Previous studies have reported variable failure rates following Rex shunt, ranging from approximately 8% to 40%, with most series reporting rates around 10–20% in pediatric populations [10]. Nevertheless, early postoperative shunt dysfunction remains a clinically relevant concern, as shunt occlusion may lead to persistent portal hypertension and recurrent variceal bleeding.

In addition to Rex shunt, alternative treatment strategies may be considered in selected patients. Transhepatic portosystemic shunts, such as transjugular intrahepatic portosystemic shunt (TIPS), may provide effective portal decompression but are associated with bypass of hepatic metabolism and an increased risk of hyperammonemia. Liver transplantation represents another definitive option, particularly in patients with unfavorable vascular anatomy or failed shunt procedures; however, it carries distinct perioperative and long-term risks [1].

However, despite the widespread adoption of staged endoscopic–surgical management, important clinical questions remain regarding the optimal timing and sequencing of interventions in children with acute portal hypertensive bleeding. In particular, evidence is limited on whether early surgical portal decompression following endoscopic stabilization improves outcomes, and whether specific subgroups, such as very young children, may have different risks of shunt dysfunction.

Therefore, this study aimed to evaluate the outcomes of a staged endoscopic–surgical strategy, with a specific focus on treatment timing (within 7 days), procedural sequencing, and the observation of age-related patterns in early Rex shunt dysfunction.

## 2. Materials and Methods

### 2.1. Ethics Statement

This study was conducted in accordance with the Declaration of Helsinki and was approved by the Institutional Review Board of the Capital Institute of Pediatrics, Capital Children’s Medical Center, Beijing, China. The requirement for additional informed consent was waived due to the retrospective design. All data were de-identified prior to analysis. Written informed consent for routine clinical care and follow-up had been obtained from the legal guardians of all patients.

### 2.2. Study Design and Patients

This was a single-center retrospective consecutive cohort study designed to evaluate the clinical outcomes of a staged endoscopic–surgical management strategy for children presenting with portal hypertension-related variceal bleeding.

Children admitted between January 2021 and December 2024 with esophagogastric variceal bleeding secondary to portal hypertension were eligible for inclusion. Clinical data were obtained from electronic medical records, endoscopy reports, imaging findings, and outpatient or telephone follow-up documentation. All eligible patients during the study period were consecutively enrolled without selective inclusion.

All patients underwent endoscopic hemostasis. Subsequent evaluation for portal decompression surgery was based on anatomical feasibility and clinical stability; therefore, not all patients proceeded to surgical intervention. This may introduce selection bias when interpreting outcomes related to surgical management.

### 2.3. Inclusion and Exclusion Criteria

The inclusion criteria were: upper gastrointestinal bleeding (hematemesis and/or melena) with endoscopic confirmation of esophageal and/or gastric varices as the bleeding source; confirmed portal hypertension-related underlying disease based on clinical and imaging evaluation (e.g., portal cavernoma, congenital hepatic fibrosis, or portal venous obstruction following biliary atresia surgery). Portal hypertension was diagnosed based on clinical features, imaging findings (such as portal cavernoma or splenomegaly), and endoscopic evidence of esophagogastric varices. Patients were also required to have received endoscopic hemostatic therapy during the index admission and to have complete clinical and follow-up data available.

The exclusion criteria were gastrointestinal bleeding unrelated to portal hypertension or incomplete endoscopic or follow-up information.

### 2.4. Peri-Procedural Management

All patients received standardized resuscitation and supportive care on admission, including fluid resuscitation, vasoactive agents (octreotide or somatostatin), and blood product transfusion when clinically indicated. Endoscopic intervention was performed after hemodynamic stabilization under general anesthesia with airway protection.

### 2.5. Endoscopic Treatment

Endoscopic hemostasis followed a standardized institutional treatment algorithm. Cyanoacrylate injection was used as first-line therapy for gastric fundal varices due to their high-flow characteristics and the technical limitations of band ligation in this setting. Esophageal variceal ligation (EVL) was added during the same session when technically feasible and clinically indicated.

Cyanoacrylate obturation was performed using N-butyl-2-cyanoacrylate according to standard practice, with injection volume and number of injection sites determined by variceal size and endoscopic appearance. EVL was performed using a multi-band ligation device, with band placement adjusted based on bleeding activity and variceal morphology.

In very young children with a narrow esophageal lumen in whom band ligation was technically difficult, cyanoacrylate injection was used as the primary hemostatic modality.

Post-procedural management included fasting, intravenous fluids, continued vasoactive therapy, and monitoring for procedure-related complications. All procedures were performed by experienced pediatric endoscopists according to institutional protocols.

### 2.6. Bridging Surgical Treatment

After successful endoscopic hemostasis and clinical stabilization, definitive surgical treatment was discussed by a multidisciplinary team including pediatric surgeons, hepatologists, and radiologists, based on portal venous anatomy, intrahepatic portal vein patency, hepatic function, and overall clinical status. As part of the staged management pathway, definitive portal decompression was generally performed within 7 days after endoscopic treatment as part of our institutional practice, aiming for early portal decompression after clinical stabilization, rather than a predefined or prospectively validated protocol.

Rex shunt was performed in patients with a patent intrahepatic left portal vein (Rex recess) suitable for physiologic portal vein reconstruction [10,11]. Splenorenal shunt was performed when Rex shunt was not anatomically feasible or when additional portal decompression was required.

Postoperative anticoagulation was routinely administered. Prophylactic unfractionated heparin was initiated immediately after surgery at a dose of 10 U/kg/h. Doppler ultrasound was performed on postoperative day 7 to assess shunt patency. In the absence of thrombosis, patients were transitioned to oral rivaroxaban according to the institutional anticoagulation protocol. No significant deviation from the institutional anticoagulation protocol occurred during the study period.

### 2.7. Outcomes and Definitions

The primary endpoints were successful endoscopic hemostasis within 72 h and the incidence of delayed rebleeding. Successful hemostasis was defined as the absence of recurrent hematemesis or worsening melena within 72 h without progressive hemoglobin decline.

Rebleeding was defined as recurrent hematemesis and/or melena requiring repeat endoscopic, surgical, or interventional treatment. Events occurring ≤5 days after initial hemostasis were classified as early rebleeding, whereas events occurring >5 days were defined as delayed rebleeding [1].

Shunt dysfunction was defined as radiologically confirmed complete occlusion or markedly reduced shunt flow on Doppler ultrasound or contrast-enhanced imaging. Early shunt dysfunction was defined as dysfunction occurring within 3 months after surgery.

Postoperative shunt patency was routinely assessed by Doppler ultrasound at 1 month, 3 months, and 6 months after surgery, and annually thereafter. Additional imaging was performed when clinically indicated.

Follow-up was conducted through scheduled outpatient visits and telephone interviews to document rebleeding events, additional interventions, and survival status. The median follow-up duration was 18 months (range 8–27 months). Follow-up was complete for all patients, with no loss to follow-up during the study period.

### 2.8. Statistical Analysis

Descriptive statistical analyses were performed using GraphPad Prism version 9.0 (GraphPad Software, San Diego, CA, USA). Continuous variables are presented as medians with ranges, and categorical variables as counts and percentages. Given the small sample size and the limited number of outcome events, the statistical analysis was primarily descriptive.

To explore the association between age group (<3 years vs. ≥3 years) and early Rex shunt dysfunction, Fisher’s exact test was applied in an exploratory manner. Because of the very small subgroup size, the results were interpreted descriptively rather than inferentially, and no formal statistical significance was assumed.

## 3. Results

### 3.1. Baseline Characteristics

A total of 12 children were included in the study. Age ranged from 1.5 to 12 years, with a median age of 6 years; two patients (16.7%) were younger than 3 years. All patients had both esophageal and gastric varices, with bleeding predominantly originating from the gastric fundus.

**Figure 1:**
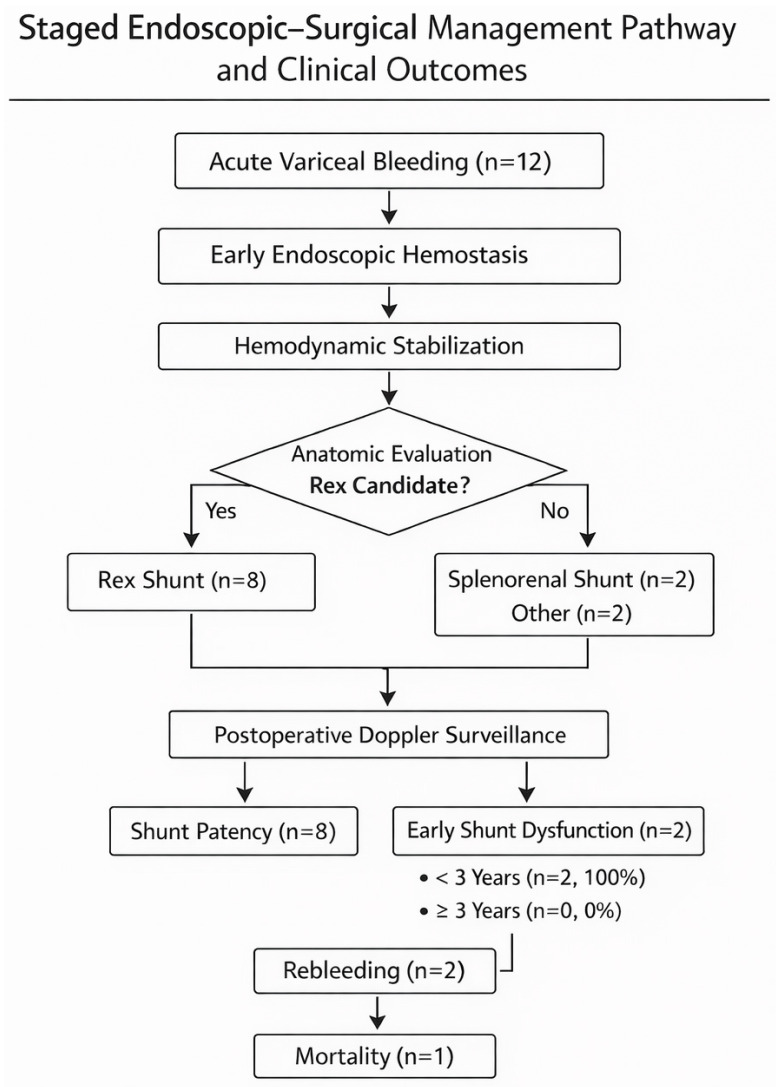
Institutional staged endoscopic–surgical management approach and observed clinical outcomes. Decision pathway illustrating the institutional management approach applied to 12 children with portal hypertension-related acute variceal bleeding. All patients underwent early endoscopic hemostasis, followed by evaluation for portal decompression when anatomically feasible. Early Rex shunt dysfunction was observed in a small number of patients younger than 3 years and was associated with subsequent rebleeding and mortality in this cohort.

Underlying etiologies included portal cavernoma in 10 patients (83.3%), congenital hepatic fibrosis in 1 patient (8.3%), and portal venous obstruction following biliary atresia surgery in 1 patient (8.3%). Baseline characteristics are summarized in Table 1. The staged endoscopic–surgical management pathway and subsequent clinical outcomes are illustrated in Figure 1.

### 3.2. Endoscopic Hemostasis and Early Outcomes

All patients underwent early endoscopic hemostasis. Combined endoscopic variceal ligation (EVL) and cyanoacrylate injection were performed in 10 patients (83.3%), while cyanoacrylate injection alone was used in 2 patients (16.7%).

**Figure 2:**
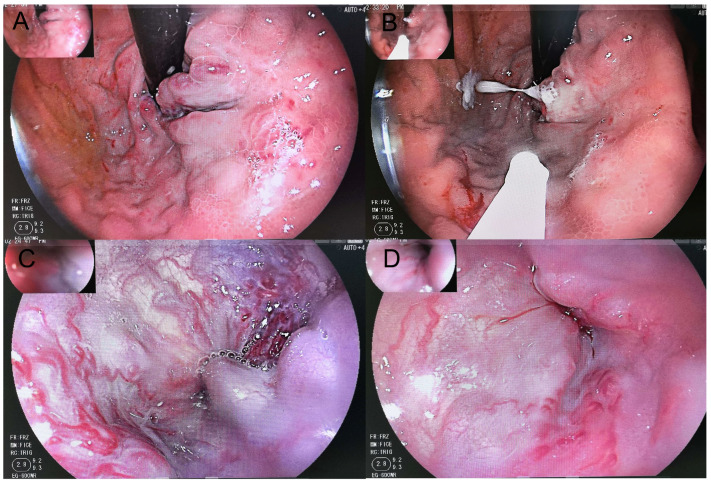
Endoscopic Findings Before and After Gastric Fundal Variceal Cyanoacrylate Injection. (**A**) Gastric fundal varices before injection. (**B**) Immediate appearance after cyanoacrylate injection. (**C**) Esophageal varices before treatment. (**D**) Immediate endoscopic observation upon withdrawal after fundal injection shows reduced tension in the esophageal varices.

Successful hemostasis within 72 h was achieved in all patients (100%), with no early rebleeding (≤5 days). Immediate post-treatment endoscopic assessment demonstrated reduced variceal tension (Figure 2).

### 3.3. Bridging Surgical Procedures

Following successful hemostasis, 10 patients (83.3%) underwent definitive portal decompression within 7 days, including 8 who underwent Rex shunt and 2 who underwent splenorenal shunt. The median interval between endoscopy and surgery was 3 days (range 2–4 days).

Two patients did not undergo bridging surgery at our center: one was transferred for liver transplantation, and one declined surgery and remained free of rebleeding during a 1-year follow-up period (Table 2).

### 3.4. Follow-Up Outcomes

The median follow-up duration was 18 months (range 8–27 months).

Among the 8 patients who underwent Rex shunt, early shunt dysfunction (<3 months) occurred in 2 of 8 patients (25%), occurring 1–3 months after surgery. Both cases occurred in children younger than 3 years. One patient developed early rebleeding at 1.2 months after surgery with confirmed shunt occlusion and was successfully managed with repeat cyanoacrylate injection followed by splenorenal shunt. The other patient experienced rebleeding at 3.0 months with shunt occlusion and subsequently died despite re-intervention. No such events were observed in patients aged ≥3 years. The overall Rex shunt patency rate during follow-up was 75% (6/8). Notably, all adverse outcomes, including rebleeding and mortality, were derived from these two events in two patients.

Rebleeding occurred exclusively in association with shunt dysfunction (2/12, 16.7%). In both patients, bleeding originated from gastric varices and was successfully controlled with repeat cyanoacrylate injection. Imaging confirmed complete Rex shunt occlusion in both patients.

Both patients subsequently underwent secondary splenorenal shunt surgery [12]. One remained shunt-patent without further bleeding, whereas the other developed recurrent shunt occlusion and died within two weeks after discharge.

Among patients who underwent definitive surgery, 8 of 10 (80%) remained free of rebleeding during follow-up.

### 3.5. Procedure-Related Complications

No severe endoscopy-related complications were observed. The rate of major complications was 0% (0/12), including ectopic embolism, gastrointestinal perforation, or severe ulcer bleeding. No additional major surgery-related complications were documented during follow-up.

## 4. Discussion

This study evaluated the outcomes of a staged endoscopic–surgical management strategy in children presenting with portal hypertension-related variceal bleeding, with particular attention to the association between young age and early Rex shunt dysfunction. Several important findings emerged. First, endoscopic therapy achieved effective early hemostasis in all patients without interval bleeding prior to surgery, supporting its role as a reliable stabilization strategy. Second, early portal decompression after endoscopic control was feasible in most patients within a short interval. Finally, early Rex shunt dysfunction occurred exclusively in children younger than 3 years, suggesting a possible age-related signal observed in a very small number of patients, which should be interpreted cautiously and requires further validation. These findings may provide preliminary insight; however, they should be interpreted cautiously given the small sample size.

Endoscopic therapy remains the cornerstone of acute management for portal hypertensive bleeding in both adults and children [9,13]. Current consensus recommendations emphasize the role of endoscopic interventions in controlling acute variceal hemorrhage and preventing early rebleeding [14]. In pediatric populations, cyanoacrylate injection has been widely used for gastric fundal varices because of their high-flow characteristics and the technical limitations of band ligation in this setting [7,8]. Previous studies and meta-analyses have reported high hemostasis rates and acceptable safety profiles for cyanoacrylate injection in gastric varices [15,16]. In our cohort, endoscopic therapy achieved successful hemostasis in all patients without early rebleeding, providing a stable window for definitive surgical evaluation. These findings support the concept that endoscopic therapy functions primarily as a bridging strategy that stabilizes acute bleeding and facilitates timely evaluation for definitive portal venous reconstruction. This staged treatment concept is consistent with current consensus recommendations. Expert pediatric opinion and the Baveno consensus emphasize that endoscopic therapy plays a key role in controlling acute variceal bleeding [17], whereas definitive management should address the underlying portal venous pathology whenever feasible, with physiologic portal reconstruction such as meso-Rex bypass considered the preferred definitive treatment in children with correctable obstruction [1,2].

Among surgical options, the meso-Rex bypass is widely considered the preferred physiologic reconstruction for extrahepatic portal vein obstruction in children because it restores hepatopetal portal flow to the liver and preserves physiological portal circulation [4]. Previous clinical series have demonstrated favorable outcomes after Rex shunt reconstruction in pediatric patients with extrahepatic portal vein obstruction [10,11,18,19]. In addition, larger cohort studies have reported encouraging medium- and long-term patency rates following Rex shunt surgery [20]. In our study, the overall Rex shunt patency rate during follow-up was 75%, which is broadly consistent with previously reported outcomes. Importantly, adverse clinical events—including recurrent bleeding and mortality—occurred only in association with shunt dysfunction, underscoring the critical importance of durable shunt patency for long-term control of portal hypertension.

A notable finding of the present study was an apparent age-related signal in early Rex shunt dysfunction. Both cases of early shunt occlusion occurred in children younger than 3 years, whereas no dysfunction occurred among older patients. Given the very small sample size, this observation should be interpreted cautiously and considered hypothesis-generating rather than indicative of a definitive pattern. Several anatomical, technical, and perioperative factors may contribute to this phenomenon, including anatomical variations, technical aspects of shunt construction, and perioperative anticoagulation strategies. Younger children typically have smaller portal venous structures, which may increase the technical complexity of vascular reconstruction and potentially predispose to early thrombosis. In addition, lower portal flow volume and altered hemodynamic conditions in smaller patients may potentially affect shunt patency. Previous studies have also suggested that anatomical and hemodynamic factors may influence surgical outcomes in children with portal venous disorders [21]. Technical factors, including graft selection and anastomotic precision, as well as perioperative anticoagulation strategies, may also play a role in early shunt outcomes. These considerations may partially explain the observed association between younger age and early shunt dysfunction.

The findings of this study have several clinical implications. A staged management strategy integrating early endoscopic stabilization with timely definitive portal decompression appears feasible in this small cohort for children presenting with acute portal hypertensive bleeding. Within this framework, heightened attention may be warranted for very young patients undergoing Rex shunt reconstruction. Early postoperative surveillance with Doppler ultrasonography and careful monitoring of shunt flow are essential for the timely detection of early shunt dysfunction.

In selected patients in which Rex shunt reconstruction is not feasible or fails early, alternative decompressive procedures such as splenorenal shunt remain important surgical options [12]. In very young children, particularly those younger than 3 years or with unfavorable vascular anatomy, liver transplantation may be considered as a potential definitive treatment option. In selected cases, transjugular intrahepatic portosystemic shunt (TIPS) may be considered as a bridging strategy, although this approach requires careful patient selection [9,22].

Several limitations should be acknowledged. First, this was a single-center study with a relatively small sample size, which limits the statistical power and generalizability of the findings. In addition, the specialized surgical expertise and institutional experience at our center may limit the generalizability of these findings to other settings. Second, the retrospective design may introduce potential selection bias despite the consecutive inclusion of patients. Third, the association between younger age and early shunt dysfunction should be interpreted cautiously and considered hypothesis-generating rather than definitive. Future multicenter studies and prospective registry-based analyses are needed to validate these findings, improve risk stratification, and optimize the timing and selection of surgical interventions in pediatric portal hypertension.

## 5. Conclusions

In this small cohort, a structured staged endoscopic–surgical strategy appears feasible for children with portal hypertension presenting with acute variceal bleeding. An age-related signal in early Rex shunt dysfunction was observed in a very small subgroup of patients, which should be interpreted cautiously and warrants further investigation in larger studies.

## Figures and Tables

**Table 1:** Overall Clinical Characteristics, Treatment, and Outcomes (n = 12).

Baseline Characteristics	
Variable	Value
Age, years, median (range)	6 (1.5–12)
Age < 3 years, n (%)	2 (16.7%)
Sex (male/female), n	5/7
Portal cavernoma, n (%)	10 (83.3%)
Congenital hepatic fibrosis, n (%)	1 (8.3%)
Post-biliary atresia surgery, n (%)	1 (8.3%)
Endoscopic Hemostasis	
Variable	Value
EVL + cyanoacrylate injection, n (%)	10 (83.3%)
Cyanoacrylate injection alone, n (%)	2 (16.7%)
Hemostasis within 72 h, n (%)	12 (100%)
Early rebleeding (≤5 days), n (%)	0
Definitive Surgical Management	
Variable	Value
Definitive surgery within 7 days, n (%)	10 (83.3%)
Rex shunt, n (%)	8 (66.7%)
Splenorenal shunt, n (%)	2 (16.7%)
No definitive surgery, n (%)	2 (16.7%)
Referred for liver transplantation	1
Declined surgery	1
Follow-up Outcomes	
Variable	Value
Follow-up duration, months, median (range)	18 (8–27)
Delayed rebleeding (>5 days), n (%)	2 (16.7%)
Time to rebleeding, months, median (range)	2.1 (1.2–3.0)
Rex shunt occlusion, n/N (%)	2/8 (25.0%)
Death, n (%)	1 (8.3%)

Note: Categorical variables are presented as n (%); continuous variables as median (range); n/N indicates proportion within the relevant subgroup denominator. All adverse outcomes (including rebleeding, shunt occlusion, and mortality) were derived from two events in two patients.

**Table 2:** Individual Patient Characteristics, Procedures, and Follow-up Outcomes.

Case	Age < 3 Years	Initial Endoscopic Therapy	Definitive Surgery	Rebleeding	Time to Rebleeding (mo)	Site	Rex Shunt Occlusion	Re-Intervention	Outcome
1	No	EVL + cyanoacrylate	Rex shunt	No	–	–	No	–	Alive
2	No	EVL + cyanoacrylate	Rex shunt	No	–	–	No	–	Alive
3	No	EVL + cyanoacrylate	Rex shunt	No	–	–	No	–	Alive
4	No	EVL + cyanoacrylate	Rex shunt	No	–	–	No	–	Alive
5	No	EVL + cyanoacrylate	Rex shunt	No	–	–	No	–	Alive
6	No	EVL + cyanoacrylate	Rex shunt	No	–	–	No	–	Alive
7	No	EVL + cyanoacrylate	Splenorenal shunt	No	–	–	–	–	Alive
8	No	EVL + cyanoacrylate	Splenorenal shunt	No	–	–	–	–	Alive
9	Yes	Cyanoacrylate only	Rex shunt	Yes	1.2	Fundus	Yes	Repeat cyanoacrylate injection → splenorenal shunt	Alive
10	Yes	Cyanoacrylate only	Rex shunt	Yes	3.0	Fundus	Yes	Repeat cyanoacrylate injection → splenorenal shunt	Died
11	No	EVL + cyanoacrylate	None	No	–	–	–	–	Alive
12	No	EVL + cyanoacrylate	Referred for LT	No	–	–	–	–	Alive

Note: ‘–’ indicates no event observed or not applicable. Shunt patency was assessed by Doppler ultrasound or contrast-enhanced imaging.

## Data Availability

All relevant data supporting the findings of this study are included in this article. Additional data are available from the corresponding author upon reasonable request.
